# Knowledge, Attitudes and Behaviours of Healthcare Workers in the Kingdom of Saudi Arabia to MERS Coronavirus and Other Emerging Infectious Diseases

**DOI:** 10.3390/ijerph13121214

**Published:** 2016-12-06

**Authors:** Abdullah J. Alsahafi, Allen C. Cheng

**Affiliations:** 1 Department of Epidemiology and Preventive Medicine, Monash University, Melbourne, VIC 3004, Australia; 2 Infection Prevention and Healthcare Epidemiology Unit, Alfred Health, Melbourne, VIC 3004, Australia; allen.cheng@med.monash.edu.au

**Keywords:** knowledge, attitudes, behaviours, practice, health care workers, MERS-CoV, emerging infectious diseases

## Abstract

***Background*:** The Kingdom of Saudi Arabia has experienced a prolonged outbreak of Middle East Respiratory Syndrome (MERS) coronavirus since 2012. Healthcare workers (HCWs) form a significant risk group for infection. ***Objectives*:** The aim of this survey was to assess the knowledge, attitudes, infection control practices and educational needs of HCWs in the Kingdom of Saudi Arabia to MERS coronavirus and other emerging infectious diseases. ***Methods*:** 1500 of HCWs from Saudi Ministry of Health were invited to fill a questionnaire developed to cover the survey objectives from 9 September 2015 to 8 November 2015. The response rate was about 81%. Descriptive statistics was used to summarise the responses. ***Results*:** 1216 HCWs were included in this survey. A total of 56.5% were nurses and 22% were physicians. The most common sources of MERS-coronavirus (MERS-CoV) information were the Ministry of Health (MOH) memo (74.3%). Only (47.6%) of the physicians, (30.4%) of the nurses and (29.9%) of the other HCWs were aware that asymptomatic MERS-CoV was described. Around half of respondents who having been investigated for MERS-CoV reported that their work performance decreased while they have suspicion of having MERS-CoV and almost two thirds reported having psychological problems during this period. Almost two thirds of the HCWs (61.2%) reported anxiety about contracting MERS-CoV from patients. ***Conclusions*:** The knowledge about emerging infectious diseases was poor and there is need for further education and training programs particularly in the use of personal protective equipment, isolation and infection control measures. The self-reported infection control practices were sub-optimal and seem to be overestimated.

## 1. Introduction

### 1.1. Background

The Kingdom of Saudi Arabia has experienced a prolonged outbreak of Middle East Respiratory Syndrome (MERS) coronavirus since 2012 [1,2]. Healthcare workers (HCWs) form a significant risk group for infection [3,4,5]. Most of the cases in health care workers occurred in the early period of the outbreak [6]. The risk of importation of other emerging infectious diseases, particularly with large population movements during the Hajj and Umrah is also significant. 

### 1.2. Aim

We aimed to explore the knowledge, attitudes and behaviours of healthcare workers in the Kingdom, particularly focusing on the recent disease of international significance MERS-coronavirus (MERS-CoV).

## 2. Methods

A survey was performed of healthcare workers in Mecca, Medina and Jeddah in the Kingdom of Saudi Arabia in 2015. The questionnaire was developed by the primary author and pilot tested on a small number of healthcare workers. Participants were recruited from 9 September 2015 to 8 November 2015. The survey was administered on paper in either Arabic or English according to respondent preference. The responses entered into an electronic database for analysis. The content areas included MERS coronavirus knowledge and sources of information; personal experiences with MERS-CoV; opinions about the location of management of patients with emerging infectious diseases; attitudes of the HCWs to infection control practices; the educational needs of the HCWs about emerging infectious diseases; and self-reported infection control practices of the HCWs. All responses were anonymous. A Chi Square test was used to compare differences in the proportions of categorical variables. Significance was determined at the 0.05 threshold. 

Ethical permission to conduct the survey was obtained from the department of medical research and studies, Jeddah, Kingdom of Saudi Arabia (approval number A00298). This department is registered in Saudi National Committee for Biomedical Ethics (Registration number H-02-J-002). 

## 3. Results

Of the 1500 invited to participate in the survey, responses were received from 1216 health care workers (HCW) included in this survey. This included 267 (22%), medical practitioners, 685 (56.5%) nurses, and 264 other healthcare workers, including health inspectors, pharmacists, lab technicians and radiology technicians. Of the participants, 472 (68.9%) of the nurses and 207 (77.5%) of the physicians working in primary health care centres. The majority of survey participants were Saudi (87.9%), and had diploma qualifications (64.5%) (Table 1).

Almost all participants had heard about MERS-CoV (98.8%) and understood it to be a problem for the community (86.1%). A significant minority (28.9%) of participants had worked at facilities where MERS-CoV had been diagnosed and many respondents had personally been tested for MERS-CoV mostly due to contact with cases within or outside the workplace (Table 1). 

### 3.1. MERS Coronavirus Knowledge and Sources of Information

Healthcare workers generally had a good understanding of the requirement to test patients admitted to ICU and those who were contacts, but a significant minority felt there was no indication for MERS-CoV investigation for the patient with acute respiratory illness requiring hospitalisation but not ICU. The majority of respondents correctly identified the need for infection prevention measures, patient risk factors and the mode of transmission by close contact. Unexpectedly, a significant proportion of respondents thought that MERS-CoV could be spread through mosquito bites. Only (47.6%) of the physicians, (30.4%) of the nurses and (29.9%) of the other HCWs were aware that asymptomatic MERS-CoV was described (Table 2). 

**Figure 1:**
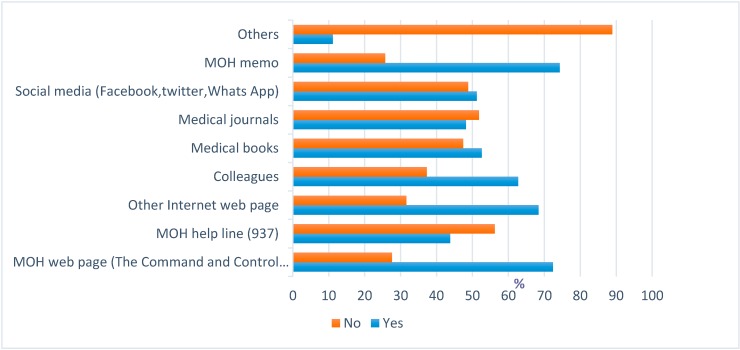
The most commonly used information sources of MERS-CoV by Healthcare workers (HCWs).

The most common sources of Middle East Respiratory Syndrome MERS-CoV information were the Ministry of Health (MOH) memo (74.3%) and MOH web page (72.4%), with smaller proportions reporting use of the MOH Helpline (937) (43.8%) and medical journals (48.2%) (Figure 1).

### 3.2. Personal Experiences with MERS-CoV

A significant minority of respondents reporting having been investigated for MERS-CoV. Only about two thirds of the HCWs (60.4%) received the result of their investigations in the first two days. It also, shows that, there are 351 (28.9%) of HCWs in this study work in places where MERS-CoV cases had been diagnosed in the last 2 years or less. (62%) of them are nurses, (21%) are physicians and (17%) are other HCWs. 145 (11.9) from the HCWs in this study were care sharing providers to MERS-CoV infected patients (Table 1).

Of these respondents, around half reported that their work performance decreased while they have suspicion of having MERS-CoV, a similar proportion had disturbances in their social lives, and almost two thirds reported having psychological problems during this period. Almost two thirds of the HCWs (61.2%) reported anxiety about contracting MERS-CoV from patients patient and more than half (56.8%) reported avoiding contact with others in public areas (Table 1). 

### 3.3. Location of Management of Patients with Emerging Infectious Diseases

A high proportion of all respondent groups felt that their workplaces were not well prepared to care for patients with emerging infectious diseases, although many respondents indicated that they were personally well prepared. 

**Figure 2:**
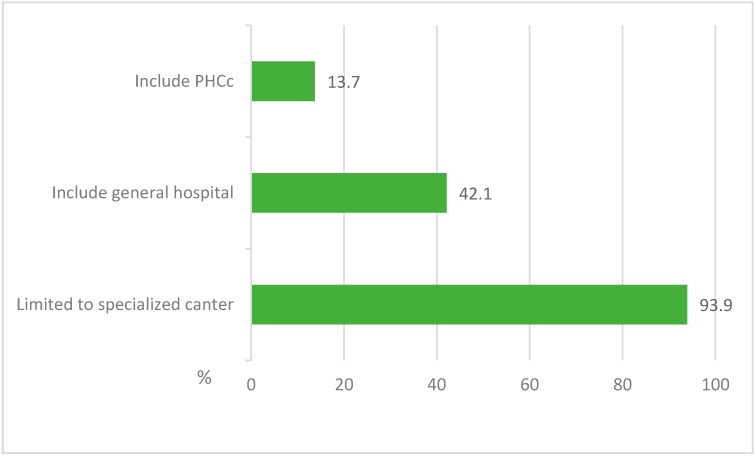
Opinions of the HCWs about the sites that should manage the MERS-CoV and other emerging infectious disease patients.

The majority of respondents believed that patients with MERS-CoV and other emerging infectious diseases should be managed in specialised centres, but a significant proportion also agreed that general hospitals also had a role in managing such patients. A minority indicated that patients with emerging infectious diseases could be managed in primary healthcare clinics (Figure 2). 

### 3.4. Educational Needs about Emerging Infectious Diseases

**Figure 3:**
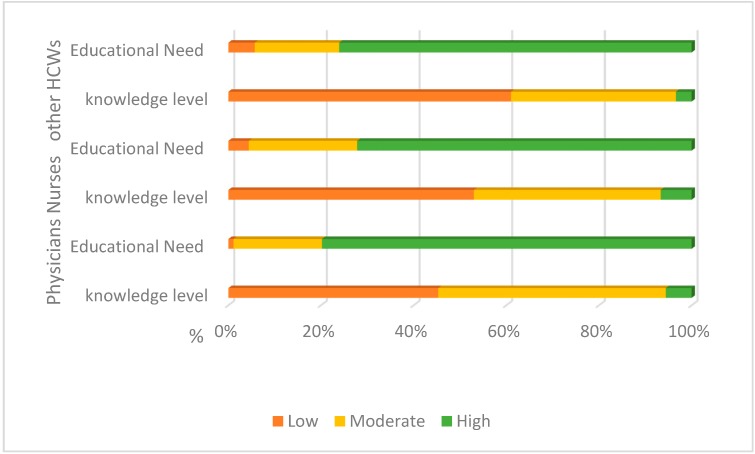
Perception of the HCWs about the level of their knowledge and the needs of educational courses about MERS-CoV and other emerging infectious diseases.

It was noted that 45% of physicians, 53% of nurses and 61% of other HCWs in the study perceive their knowledge about MERS-CoV, Ebola and others emerging infectious diseases to be low, while 40% of them indicated that it was moderate and ≤7% indicated it was high (Figure 3).

As expected, the majority of the HCWs in the study (≥72.3%) indicated that that they are in need for educational courses and training about the MERS-CoV, Ebola and other emerging infectious diseases (Figure 3).

### 3.5. Attitudes to Infection Control Practices

A large majority of participants reported that they were more eager to apply infection control measures since the onset of MERS-CoV in KSA. Unexpectedly, almost two thirds of respondents were unaware of guidelines or protocols for the care of patients with MERS-CoV infection. Only 22.8% reported having received training about dealing with infectious disease outbreaks, 37.1% reported training in infection control policies and procedures, 54.4% reported training in hand hygiene and 45.6% reported training in N95 mask wearing techniques (Table 3). 

A high proportion of respondents agreed that emergency department overcrowding, poor hand hygiene and mask use contributed to the risk of HCW being infected with MERS-CoV. Similarly, a high proportion agreed that a lack of knowledge about the mode of transmission, a lack of policies and procedures, and insufficient training in infection control procedures also contributed to the risk (Table 3). 

### 3.6. Self-Reported Infection Control Practices

Self-reported compliance with hand hygiene was moderate, with only about two thirds of the HCWs (60.3%) of the physicians, (64.8%) of the nurses and (60.6%) of the other HCWs practicing regular hand washing after patient contact. Less than half of respondents reported full compliance with use of surgical masks when required, and a similar proportion reported compliance with N95 respirators when required (Table 3). 

Compliance with immunisation recommendations was poor, with only 59.5% self-reporting receipt of annual influenza vaccine within the last 12 months, 74.4% reporting receipt of meningococcal vaccine in the last 3–5 years, and 50.4% reporting have received hepatitis B immunisation or testing for immunity during their work career (Table 3).

## 4. Conclusions

The control of emerging infectious diseases in the hospitals can be limited by case detection and management using transmission-based precautions to all confirmed and probable cases. For MERS-CoV in health care settings, this requires early recognition, testing and airborne precautions [7]. In this survey we found that despite a high basic level of awareness about MERS coronavirus and the importance of infection control, there remained significant misconceptions. We have previously described more than 171 secondary cases in healthcare workers in the 939 cases reported to July 2015 with another 174 cases acquired by other patients while in hospital [8]. Another study suggested that, infected health care workers were an important group involved in disease spread [9]. 

This survey revealed that, about two third of the HCWs whose contact to MERS-CoV cases were investigated for possible infection, which may reflect a high index of suspicion , the anxiety about infection and accessibility to health services. This study also showed significant proportion with personal experience of MERS-CoV either as HCW at institutions caring for cases or being investigated for possible infection following contact with cases [10].

A survey of healthcare workers in South Korea found a poor level of knowledge of the modes of transmission, which was implicated in the rapid spread of the infection in hospitals. Worryingly, more than half of respondents in this survey thought that MERS-CoV could be spread through mosquito bites [10]. The infection control measures are very crucial for respiratory infectious cases in the healthcare institutes [11]. A high proportion of respondents identified hospital overcrowding, poor hand hygiene and mask use, lack of knowledge about the mode of transmission, a lack of policies and procedures, and insufficient training in infection control procedures also contributed to the risk of spread. Self-reported adherence with infection control measures was surprisingly poor, particularly in light of previous studies suggesting that self-reported adherence generally overestimates observed behaviour. 

The results of this survey suggest that there was poor knowledge about emerging infectious diseases, and self-reported infection control practices were sub-optimal. However, there was recognition in respondents of the need for further education and training, particularly in the use of personal protective equipment despite the high level of trust in official sources of information. System level improvements, such as incorporation of emerging infectious diseases into medical schools and continuous medical education programs, the implementation of isolation and infection control measures, and appropriate nursing-to-patient ratios would also improve preparedness [12]. 

## Figures and Tables

**Table 1:** Socio-demographic characteristics of participants and their awareness about Middle East Respiratory Syndrome Coronavirus (MERS-CoV).

Socio-Demographic Fetchers	Physicians	Nurses	Other HCWs *	Total	
	*N* (%)	*N* (%)	*N* (%)		
Number	267 (22)	685 (56.3)	264 (21.7)	1216 (100)	
Gender:					
Male	199 (74.5)	299 (43.6)	201 (76.1)	699 (57.5)	
Female	68 (25.5)	386 (56.4)	63 (23.9)	517 (42.5)	
Work place:					
Hospital	57 (21.3)	204 (29.8)	31 (11.7)	292 (24)	
PHC	207 (77.5)	472 (68.9)	228 (86.4)	907 (74.6)	
Other	3 (1.1)	9 (1.3)	5 (1.9)	17 (1.4)	
Have you heard about Middle East Respiratory Syndrome (MERS)?					*p* **
Yes	263 (98.5)	677 (98.8)	261 (98.9)	1201	0.906
No	4 (1.5)	8 (1.2)	3 (1.1)	15	
MERS has been diagnosed in patients in the practice of the HCW:					
Yes	75 (28.10)	218 (31.8)	58 (22)	351	0.02
No	192 (71.9)	462 (67.4)	203 (76.9)	857	
Don’t Know	0 (0)	5 (0.7)	3 (1.1)	8	
Do you think that Middle East Respiratory Syndrome (MERS) a problem in this community?					
Strongly agree	118 (44.2)	362 (52.8)	145 (54.9)	625	0.105
Agree	104 (39)	230 (33.6)	88 (33.3)	422	
Neutral	24 (9)	65 (9.5)	18 (6.8)	107	
Disagree	17 (6.4)	23 (3.4)	10 (3.8)	50	
Strongly disagree	4 (1.5)	5 (0.7)	3 (1.1)	12	
The HCWs who had been investigated for MERS-CoV and the duration from sample taking and releasing the result:					
One day	7 (23.3)	54 (36.7)	18 (45)	79	0.013
2 days	10 (33.3)	34 (23)	8 (20)	52	
3 days	9 (30)	46 (31.2)	9 (22.5)	64	
More	4 (13.3)	13 (8.8)	5 (12.5)	22	
The impact of suspicion of having MERS-CoV on the HCWs work performance, social and psychological life:					
Work performance:	17 (17.7)	61 (63.5)	18 (18.8)	96	0.006
Social life	18 (16.1)	72 (64.3)	22 (19.6)	112	0.001
Psychological life	18 (13.3)	96 (71.1)	21 (15.6)	135	0.001
Clinical experience of the HCWs in the last 2 years or less regarding:					
Working in place where MERS-CoV infected patient was diagnosed or admitted.	75 (28.1)	218 (31.8)	58 (22)	351	0.02
Cared a MERS-CoV infected patient.	24 (9)	106 (15.5)	15 (5.7)	145	<0.001
After the MERS-CoV outbreak:					
Sometimes get scared of going to work for fear of contacting MERS-CoV patient.	123 (46.1)	448 (65.4)	173 (65.5)	744	<0.001
Sometimes avoid body contact whenever HCWs are in a public area.	116 (43.4)	421 (61.5)	154 (58.3)	691	<0.001

* Healthcare Workers; ** *p* values represent results of chi-squared test for the null hypothesis of no difference in the Socio-demographic characteristics of participants and their awareness about MERS-CoV.

**Table 2:** Health care workers (HCWs)’ knowledge about MERS-CoV infection.

Knowledge about MERS-CoV Infection	Correct Responses				*p* **
	Physicians	Nurses	Other HCWs *	Total	
	*N* (%)	*N* (%)	*N* (%)		
**Indications of testing for MERS-CoV:**					
Acute respiratory illness requiring ICU	224 (83.9)	552 (80.6)	188 (71.2)	964	<0.001
Acute respiratory illness requiring hospitalisation but not ICU	153 (57.3)	391 (57.1)	154 (58.3)	698	<0.001
Mild respiratory illness not requiring hospitalisation	170 (63.7)	316 (46.1)	108 (40.9)	594	<0.001
Mild acute respiratory illness where there is a history of contact with a confirmed MERS case	203 (76)	409 (59.7)	175 (66.3)	787	<0.001
Other acute non-respiratory illness in patients with a history of contact with a confirmed MERS case	189 (70.8)	437 (63.9)	153 (58.2)	779	<0.001
**MERS-CoV infection:**					
About 3–4 out of every 10 people reported with MERS-CoV have died.	149 (55.8)	345 (50.4)	121 (45.8)	615	<0.001
Some infected people had mild symptoms (such as cold-like symptoms)	212 (79.4)	441 (64.4)	146 (55.3)	799	<0.001
Most of the people who died had an underling medical condition	196 (73.4)	407 (59.4)	149 (56.4)	752	<0.001
MERS-CoV has spread from ill people to others through close contact	233 (87.3)	530 (77.4)	189 (71.6)	952	<0.001
The possibility of transmission through infected camel and bats	194 (72.7)	340 (49.6)	122 (46.2)	656	<0.001
Higher risk for getting MERS-CoV or having a severe case include pre-existing conditions such as diabetes; cancer, renal failure and patients taking immunosuppressive drugs.	235 (88)	476 (69.5)	165 (62.5)	876	<0.001
The incubation period for MERS is usually about 5 or 6 days but can be more	183 (68.5)	333 (48.6)	100 (37.9)	616	<0.001
MERS is spread through mosquito bite	159 (59.6)	284 (41.50)	99 (37.5)	542	<0.001
Some infected people had no symptoms	127 (47.6)	208 (30.4)	79 (29.9)	414	<0.001
MERS-CoV infected patient need isolation	253 (94.8)	616 (89.9)	228 (86.4)	1097	<0.001

* Healthcare Workers; ** *p* values represent results of chi-squared test for the null hypothesis of no difference in Correct responses of the HCWs knowledge about MERS-CoV infection across groups.

**Table 3:** HCWs attitudes and barriers to infection control practices following MERS-CoV outbreak.

Self-Reporting Infection Control Practice	Physicians	Nurses	Other HCWs *	Total	*p* **
	*N* (%)	*N* (%)	*N* (%)		
**Hand washing after patient contact:**					
Always	161 (60.3)	444 (64.8)	160 (60.6)	765	0.040
Very Often	69 (25.8)	130 (19)	50 (18.9)	249	
Sometimes	33 (12.4)	96 (14)	40 (15.2)	169	
Rarely	3 (1.1)	11 (1.6)	12 (4.5)	26	
Never	1 (0.4)	4 (0.6)	2 (0.8)	7	
**Wearing of surgical mask during patient contact:**					
Always	115 (43.1)	308 (45)	106 (40.2)	529	<0.001
Very Often	75 (28.1)	157 (23)	58 (22)	290	
Sometimes	60 (22.5)	162 (23.6)	51 (19.3)	273	
Rarely	14 (5.2)	44 (6.4)	32 (12.1)	90	
Never	3 (1.1)	14 (2)	17 (6.4)	34	
**Wearing of N95 mask during patient contact:**					
Always	118 (44.2)	317 (46.3)	105 (39.8)	540	0.009
Very Often	57 (21.4)	136 (19.9)	53 (20.1)	246	
Sometimes	38 (14.2)	129 (18.8)	51 (19.3)	218	
Rarely	35 (13.1)	76 (11.1)	27 (10.2)	138	
Never	19 (7.1)	27 (3.9)	28 (10.6)	74	
**After the MERS outbreak, are the HCWs become more eager to apply infection control measures?**					
Strongly Agree	184 (68.9)	429 (62.6)	162 (61.4)	775	0.12
Agree	72 (27)	202 (29.5)	78 (29.5)	352	
**Do you have a guideline or protocol for caring for patients with MERS?**					
Yes	142 (53.2)	234 (34.2)	65 (24.6)	441	<0.001
No	62 (23.2)	238 (34.7)	98 (37.1)	398	
Don’t Know	63 (23.6)	213 (31.1)	101 (38.3)	441	
**HCWs training assessment:**					
How to deal with an infectious disease outbreak.	80 (30)	152 (22.1)	45 (17.8)	277	0.011
Infection control policies and procedures.	98 (36.7)	285 (41.6)	68 (25.8)	451	<0.001
Hand washing techniques.	158 (59.2)	389 (56.8)	115 (43.6)	662	0.001
N95 mask wearing techniques.	145 (54.3)	317 (46.3)	92 (34.8)	554	<0.001
IN the last 12 months, the HCW who got an annual influenza vaccine.	182 (68.2)	403 (58.8)	138 (52.3)	723	0.001
IN the last 3–5 years, the HCW who got Meningitis vaccine.	209 (78.3)	502 (73.3)	194 (73.5)	905	0.263
HCW through his work career who ever had been took viral hepatitis (B) immunisation or examined for its antibodies.	172 (64.4)	331 (48.3)	110 (41.7)	613	<0.001
**The barriers to infection control practices as addressed by HCWs**					
Lack of knowledge about the mode of transmission of the disease MERS-CoV:	240 (90)	645 (94.2)	238 (90.2)	1123	0.118
Not wearing mask while examine or contact with the patient:	262 (98)	647 (94.5)	252 (95.5)	1161	<0.001
No hand washing after examine or contact with the patient:	260 (97.3)	652 (95.2)	250 (94.7)	1162	<0.001
Limitation of infection control material:	217 (81.3)	603 (88)	213 (80.7)	1033	0.014
Lack of policy and Procedures in infection control:	227 (85)	598 (87.3)	236 (89.4)	1061	0.003
Insufficient training in infection control measurements:	243 (91)	633 (92.4)	240 (90.9)	1116	0.178
Less commitment of health care workers to the policies and procedures:	250 (93.6)	659 (96.2)	242 (98.4)	1151	<0.001
Overcrowding in ER:	258 (96.6)	666 (97.2)	246 (93.2)	1170	0.010

* Health Care Workers; ** *p* values represent results of chi-squared test for the null hypothesis of no difference in HCWs attitudes and barriers to infection control practices following MERS-CoV outbreak between HCWs groups.
